# Internet Use and Self-Rated Health Among Older Adults: Scoping Review

**DOI:** 10.2196/76930

**Published:** 2026-02-19

**Authors:** Hanan AboJabel, Fareeda Abo-Rass

**Affiliations:** 1 The Paul Baerwald School of Social Work and Social Welfare Hebrew University of Jerusalem Jerusalem Israel; 2 Department of Social Work Ben-Gurion University of the Negev Beer-Sheva Israel

**Keywords:** internet use, older adults, scoping review, self-rated health, social participation

## Abstract

**Background:**

Self-rated health (SRH) is a robust predictor of morbidity, functional decline, and mortality in later life. As internet use becomes increasingly embedded in older adults’ daily routines, clarifying its association with SRH and the pathways through which it may operate is important for research, practice, and policy.

**Objective:**

This scoping review aimed to map and characterize the international evidence on the association between internet use and SRH among older adults, synthesize how potential mediators and moderators have been examined, and identify key methodological, theoretical, and population gaps in the literature.

**Methods:**

Guided by the Joanna Briggs Institute methodology and PRISMA-ScR (Preferred Reporting Items for Systematic Reviews and Meta-Analyses extension for Scoping Reviews) reporting standards, we conducted a scoping review and searched 5 databases: PubMed, CINAHL, AgeLine, PsycINFO, and Web of Science. The final search was performed on February 5, 2024. Reference lists were screened, and Google Scholar searches were conducted as supplementary search methods.

**Results:**

Database searches identified 4294 records; after removing 615 duplicates, 3679 records were screened, and 77 full texts were assessed, resulting in 27 included studies. All included studies were quantitative, and the evidence base was predominantly cross-sectional (25/27). Explicit theoretical frameworks were used in 6 out of 27 studies. Most studies were published between 2019 and 2024 (22/27) and were conducted most frequently in China (11/27) and the United States (7/27). All studies were conducted in high-income countries. SRH was typically assessed using a single-item measure, while internet use was operationalized as access/use (yes/no), frequency, and/or purpose/domain-specific measures. Most studies reported a statistically significant positive association between internet use and better SRH (24/27), with socially oriented uses (eg, communication and social participation) showing the most consistent associations. Mediating pathways were examined in 6 out of 27 studies, and most often suggested social mechanisms such as greater social support, higher social engagement, and lower loneliness. Subgroup heterogeneity was reported in 10 out of 27 studies, including differences by age, gender, residence, and marital status.

**Conclusions:**

Overall, internet use, particularly socially oriented use, was most consistently associated with better SRH among older adults. Policy efforts should support digital inclusion by improving access, skills, and ongoing assistance that enable meaningful use for social connection and service access. At the same time, nondigital options are essential to avoid excluding older adults who do not use the internet. In addition, evidence gaps, including limited use of theoretical frameworks and scarce data from low- and middle-income countries, underscore the need for theory-informed longitudinal and intervention studies to strengthen causal inference, expand knowledge on mediating and moderating factors, and assess generalizability across diverse contexts.

## Introduction

Self-rated health (SRH) is a measure of individuals’ subjective assessments of their general health, encompassing physical, mental, social, and cultural aspects [1-3]. Usually, SRH is assessed using a single item, often phrased as: “Would you say your general health is excellent, very good, good, fair, or poor?” [4]. This scale is widely used to assess health status among older adults, as it is considered valid, reliable, quick, and easy to administer [5,6]. Indeed, SRH is regarded as an important predictor of healthy aging, future morbidity, functional capacity, hospitalization, health care consumption, and mortality [7-11]. The literature indicates that a variety of factors may play an important role in good SRH among older adults, including background characteristics (eg, younger age, higher education, and male gender), social support networks, good mental and physical health, the ability to care for oneself, and healthy behaviors (eg, sufficient physical activity) [1,12-17].

An additional factor that may be related to older adults’ SRH is internet use. The internet offers **a range of opportunities** to promote independent living among older adults and to improve their quality of life and overall well-being [18-21]. It facilitates communication and helps older adults maintain relationships with family and friends, as well as establish new social connections [19,21]. In addition, the internet enables participation in online leisure activities [22,23]. Furthermore, older adults can use the internet to seek information, including health-related information, potentially increasing health literacy, and supporting better health-related decision-making [24-26]. However, extensive internet use also entails potential risks. For instance, frequent internet use for nonsocial purposes, such as information seeking and entertainment, may reduce social interactions and weaken existing social networks, thereby increasing social exclusion [27-29]. There is also a risk of internet addiction among older adults [30], along with exposure to privacy invasion and data theft [31,32]. These opportunities and risks may, in turn, influence older adults’ SRH.

In recent years, a growing body of research has examined the association between internet use and SRH among older adults; however, this literature has not yet been comprehensively synthesized and organized. Conducting a scoping review is therefore warranted, given the increasing integration of the internet into later life and the expansion of online services, especially in health and social domains [33-35]. In addition, digital technologies have been suggested as potentially contributing to responses to population aging and to alleviating pressures on health systems [36,37].

Therefore, the aim of this scoping review is to map and synthesize the existing international literature on internet use and SRH in later life by (1) describing how internet use and SRH have been conceptualized, operationalized, and examined across studies, as well as mapping the reported patterns of findings regarding the direction and nature of the association between internet use and SRH; (2) mapping how the literature addresses potential pathways linking internet use and SRH, including mediating and moderating factors, where mediators are defined as variables that explain the mechanisms through which one variable influences another, and moderators are defined as variables that affect the strength or direction of this relationship [38]; and (3) identifying methodological and theoretical gaps, as well as underrepresented populations within the existing evidence base. Identifying these elements is essential for informing the development of evidence-based interventions aimed at promoting SRH and well-being in later life through the use of digital technologies, as well as for supporting the design and advancement of policies in the fields of aging and digital health.

## Methods

### Overview

This scoping review was conducted in accordance with the Joanna Briggs Institute (JBI) methodology for scoping reviews and is reported following the PRISMA-ScR (Preferred Reporting Items for Systematic Reviews and Meta-Analyses extension for Scoping Reviews) [39]. In line with scoping review methodology, no formal appraisal of methodological quality was conducted [39]. An overview of adherence to the PRISMA-ScR reporting criteria is presented in Multimedia Appendix 1.

### Search Strategy

The search strategy was reported in accordance with the PRISMA-S (Preferred Reporting Items for Systematic Reviews and Meta-Analyses extension for Searching) guideline to enhance transparency and reproducibility [40]. An overview of adherence to the PRISMA-S reporting criteria is provided in Multimedia Appendix 2. The search strategy was developed by the first author, who has expertise in aging and technology, and reviewed by the second author. It was developed iteratively, guided by the review objectives, key concepts identified in the literature, and preliminary exploratory searches. The search was conducted across 5 databases relevant to the research topic: PubMed, CINAHL, AgeLine, PsycINFO, and Web of Science. Searches were limited to peer-reviewed journal articles published in English. The final search was performed on February 5, 2024, and included all publications available up to that date. The search strategy combined keywords and Boolean operators representing 3 core concepts: internet use (“Internet” or “web” or “online” or “digital” or “technology” or “information and communication technology” or “social media” or “computer” or “smartphone” or “tablet”), SRH (“self-rated health” or “self-reported health” or “self-assessed health” or “perceived health” or “subjective health”), and older adults (“older adults” or “aging” or “aged” or “older people” or “elderly” or “seniors”). Full search strategies for each database are presented in Multimedia Appendix 3. To enhance the comprehensiveness of the search, reference lists of all included articles were screened, and Google Scholar was used as a supplementary search tool, consistent with recommendations to use Google Scholar as a complementary resource in systematic and scoping reviews rather than as a stand-alone database [41]. No additional eligible studies were identified through these supplementary searches. Duplicates were removed manually by the first author by comparing titles, authors, and publication details across records, and the deduplication was verified by the second author.

### Inclusion and Exclusion Criteria

Studies were eligible for inclusion if they met the following criteria: (1) the study reported original empirical findings derived from quantitative, qualitative, or mixed methods research, based on either primary data collection or secondary data analysis; (2) the study explicitly addressed the conceptualization and/or operationalization of the key constructs and examined the relationship between internet use and SRH; (3) the study population consisted primarily of older adults, defined as individuals aged 60 years and older, or was explicitly described by the original authors as comprising older adults, in line with commonly used definitions in previous systematic and scoping reviews (Huang et al [42]). Studies with broader age ranges were also eligible if results for older adults were reported separately; (4) the article was published in English; and (5) the article appeared in a peer-reviewed journal. Studies were excluded if they were opinion pieces, conference abstracts, book reviews, book chapters, theses, or doctoral dissertations. In addition, studies in which internet use was modeled as the outcome variable and SRH as the independent variable were excluded. This decision ensured methodological consistency by maintaining a uniform analytical direction, as is common in systematic and scoping reviews [43,44], thereby facilitating more coherent evidence mapping and synthesis across studies.

### Data Charting

Data charting was guided by a structured framework informed by the JBI data extraction template for scoping reviews and adapted to the objectives of this review [45]. Data were charted from each included article at the individual-study level, including bibliographic details, study aims, study design, stated theoretical framework, sample characteristics, and the measures used to assess internet use and SRH. Key findings were also charted, including the reported association between internet use and SRH and mediators or moderators explicitly examined or discussed by the study authors. The first author conducted the data charting manually by reviewing each included article in full. To enhance reliability, the second author reviewed the charted data and the data charting framework for accuracy and consistency, and uncertainties were resolved through discussion and consensus.

### Data Analysis and Synthesis

Prior to finalizing the approach for analysis and presentation, the authors conducted a preliminary review of 5 randomly selected included articles to inform the structure and focus of the subsequent analysis. Building on the charted data, study characteristics were organized descriptively to map the evidence base and to support a systematic presentation of study characteristics in the Results section (Table 1). Subsequently, a narrative synthesis of the reported findings was conducted to identify recurring patterns across studies. The synthesis summarized the direction of the association between internet use and SRH and organized results according to key dimensions aligned with the review objectives, including the type of internet use and variation across population subgroups. Findings related to mediating pathways and between-study variation were integrated where reported, to support a coherent presentation of results in the Results section, while Table 2 provided a concise overview of the synthesized evidence. To ensure the reliability of the analytical process, both authors jointly reviewed the organization and presentation of the synthesized findings, and any discrepancies were resolved through discussion and consensus.

**Table 1:** Characteristics of included studies.

Authors (country)	Aims	Study design/theoretical framework	Sample characteristics	Measure of SRH^a^	Measure of internet use
Chen et al 2022 [46] (China)	To examine the association between internet use and health outcomes, including the mediating role of cultural engagement	Cross-sectional; Chinese General Social Survey (2015 and 2017 waves) Activity theory (Fernández-Ballesteros et al 2021 [47])	N=6066 older adults aged ≥60 years; mean age 69.19 (SD 7.27) years; 52% women; mean education 1.5492 (SD 0.6378); range 1 (“primary school”) to 3 (“college or above”)	Single item (unhealthy/healthy)	Internet use (yes/no)
Chopik 2016 [48] (United States)	To examine the association between socially oriented technology use and health-related outcomes	Cross-sectional; Health and Retirement Study (2012 wave)	N=591 older adults; mean age 68.18 (SD 10.75) years; 55.5% women; mean education 13.28 (SD 2.70) years; 73.9% White, 19.9% Black/African American, 6.1% Hispanic	Single item (5-point scale: 1 “poor” to 5 “excellent”)	Social technology use was measured using 5 modalities (email, social networking sites, online video/phone calls, online chat/instant messaging, smartphone); summed index (0-5)
Ding et al 2023 [49] (China)	To examine the association between internet access and health-related outcomes	Cross-sectional; China Health and Retirement Longitudinal Study (2011, 2013, 2015, and 2018 waves)	N=57,960 older adults; mean age 58.7 (SD 8.09) years; 50% women; mean education 1.712 (SD 1.304)	Single item (poor health/good health)	Internet access (yes/no)
Duplaga et al 2021 [50] (Poland)	To examine the association between internet use and health-related outcomes	Cross-sectional; telephone survey	N=1000 adults aged ≥50 years; 39.3% aged 50-59 years, 28.5% aged 60-69 years, 29.7% aged ≥70 years; 55.8% women; education: 27.8% below secondary, 45.9% secondary/postsecondary, 26.3% university	Single item (“unsatisfactory”/”at least satisfactory”)	Frequency of internet use (4-point scale: 0 “no use,” 1 “a few times a month or less,” 2 “a few times a week,” 3 “every day”)
Falk Erhag et al 2019 [51] (Sweden)	To examine the association between internet use and SRH	Cross-sectional; Gothenburg H70 Birth Cohort Study (2014-2016 wave); personal interview and self-administered questionnaire	N=1136 older adults aged 70 years; 69.3% men; 82.9% had >9 years of education; 28.5% had a university degree	Single item (5-point scale: “excellent” to “poor”)	Frequency of internet use (7-point scale: “never” to “daily or several times a day”)
Fjell et al 2020 [52] (Norway)	To examine the association between SRH and factors related to demographics, lifestyle, health conditions, and medical diagnoses	Cross-sectional; face-to-face interview	N=233 older adults participating in a preventive home visit program; mean age 77 (range 75-79) years; 50% women; education: 25% >7 years, 75% ≤7 years	Single item (5-point scale: 1 “poor” to 5 “excellent”)	Internet use (yes/no)
Gracia and Herrero 2009 [53] (Spain)	To examine the association between internet use and SRH	Cross-sectional; telephone/online survey (Digital divide and quality of life among older people)	N=709 adults aged ≥55 years; 56.7% aged 55-64 years, 43.3% aged 65-74 years; 52.2% women; social class: 8.1% high, 9.2% medium-high, 26.3% medium, 35.3% medium-low, 21% low	Single item (good health/poor health)	Internet use (user/nonuser)
Jeon and Choi 2024 [54] (Korea)	To examine the association between internet use and physical and psychological health outcomes	Cross-sectional; Living Profiles of Older People Survey (2020 wave); face-to-face interviews	N=5094 older adults aged ≥65 years; mean age 70.71 (SD 2.8) years; 54.1% women; education: 9.7% college+, 42.6% high school, 25.3% middle school, 19.3% elementary school, 3.1% no formal education	Single item (5-point scale: 1 “very good” to 5 “very poor”)	Internet use: Interpersonal communication: 3 yes/no items; summed index (0-3) Instrumental use: 8 yes/no items; summed index (0-8)
Kim et al 2020 [55] (United States)	To examine the association between ICT^b^ use and health-related outcomes	Cross-sectional; National Health and Aging Trends Study (2010 wave)	N=4976 older adults aged ≥65 years; mean age 73.7 (SD 0.09) years; 54.3% women; mean education 2.8 (SD 0.4); range 1 (“less than 12th grade”) to 5 (“graduate degree”); 83.5% White, 7.6% Black, 2.9% other	Single item (5-point scale: 1 “poor health” to 5 “excellent health”)	ICT: IT (6 items; yes/no): 3 items for personal tasks >3 items for health-related information. Communication technology (1 item; frequency): sending messages by email or texting (rarely/some days/most days).
Koopman-Boyden and Reid 2009 [56] (New Zealand)	To examine the association between internet use and well-being	Cross-sectional; phone/online survey	N=1680 older adults aged 65-84 years; 31% aged 65-69 years, 28% aged 70-74 years, 25% aged 75-79 years, 16% aged 80-84 years	Single item (satisfied/dissatisfied with health)	Internet use (yes/no)
Lee and Jang 2022 [57] (Korea)	To examine the association between changes in internet use during the COVID-19 pandemic and SRH, comparing young-old (65-74) and old-old (≥75) adults	Cross-sectional; Digital Divide Survey (2020 wave); face-to-face interviews	N=1150 older adults aged ≥65 years: young-old (n=670; mean age 69.18 (SD 2.8) years; 52.5% women; 23.4% <middle school, 76.6% ≥high school) and old-old (n=480; mean age 79.93 (SD) 3.6 years; 62.9% women; 44.8% <high school, 55.2% ≥high school)	Single item (4-point scale: 1 “not satisfied at all” to 4 “very satisfied”)	Internet use: 4 domains (each rated on a 5-point scale from 1 “significantly decreased” to 5 “significantly increased”): (1) social networking and information-sharing services; (2) social participation services; (3) daily services; and (4) search, email, and content services
Lee et al 2018 [58] (United States)	To examine the association between ICT use and health-related outcomes among older cancer survivors	Cross-sectional; National Health and Aging Trends Study (2011 wave)	N=1411 community-dwelling cancer survivors aged ≥65; 53.6% aged 65-74 years, 36.2% aged 75-84 years, 10.2% aged ≥85 years; 48% women; mean education 2.9 (SD 0.22); range 1 (“less than 12th grade”) to 5 (“graduate degree”); 90% White, non-Hispanic	Single item (5-point scale: 1 “poor” to 5 “excellent”)	ICT use: Communication technology: Frequency of emailing/texting (no or rarely/some days/most days) Information technology: 6 yes/no items, summarized as 2 indices: personal tasks (3 items) and health-related internet use (3 items)
Li et al 2023 [59] (China)	To examine the association between online and offline social activities and health-related outcomes	Cross-sectional; World Values Survey (wave 7, China sample) and China Health and Retirement Longitudinal Study (2018 wave)	Sample 1: N=598 older adults aged ≥60 years; mean age 64.76 (SD 2.97) years; 52.7% women; education: 53.8% primary school or below, 26.4% middle school, 15.6% high school/vocational, 3.8% bachelor’s, 0.3% master’s or above. Sample 2: N=9434 older adults; mean age 68.53 (SD 6.44) years; 50% women; education: 73.7% primary school or below, 16.3% middle school, 8.5% high school/vocational, 0.7% associate degree, 0.6% bachelor’s, 0.03% master’s or above	Single item (5-point scale: 1 “very poor” to 5 “very good”) in both samples	Sample 1: Frequency of internet use (5-point scale: 1 “never” to 5 “daily”). Sample 2: Frequency of internet use (0-3 scale: 0 “no participation” to 3 “almost daily”; past month)
Liu et al 2023 [60] (China)	To examine the association between internet use and SRH, including the mediating role of social engagement and heterogeneity by living arrangements	Cross-sectional; China Health and Retirement Longitudinal Study (2018 wave)	N=14,587 middle-aged and older adults aged ≥45 years; mean age 61 (SD 9.248) years; 52% men; education: 62% elementary school or below, 23% junior high, 13% high school or above	Single item (5-point scale: 1 “very poor” to 5 “very good”)	Two measures: Internet use (yes/no) Frequency of internet use (4-point scale: 0 “never” to 3 “almost every day”)
Liu et al 2022 [61] (China)	To examine the association between internet use and SRH, including the mediating role of social support	Cross-sectional; Chinese General Social Survey (2017 wave)	N=4234 older adults aged ≥60 years; mean age 69.34 (SD 7.37) years; 48% men; mean education 6.65 (SD 4.61) years	Single item (5-point scale: 1 “very unhealthy” to 5 “very healthy”)	Internet access (yes/no)
Lyu and Sun 2021 [62] (China)	To examine the association between internet use and SRH, including the mediating role of social capital	Cross-sectional; China Family Panel Studies (2018 wave)	N=7193 older adults aged ≥60 years; age distribution: 64.83% aged 60-69 years, 28.69% aged 70-79 years, 6.48% aged ≥80 years; 51.01% men; education: 88.39% had 0-9 years, 10.86% had 10-15 years, 0.75% had ≥16 years	Single item (unhealthy/healthy)	Internet use (yes/no)
Millar et al 2020 [63] (United States)	To examine the association between problem-solving in technology-rich environments and SRH, focusing on age differences	Cross-sectional; Program for the International Assessment of Adult Competencies (2012-2014 wave) Paasche-Orlow and Wolf’s [64] model (2007) and Gewald and Rockmann’s [65] model (2016)	N=3260 adults aged ≥35 years; age: 26.65% 35-44 years, 28.71% 45-54 years, 27.12% 55-65 years, 16.58% ≥66 years; 54.82% women; education: 60.74% high school or less, 39.25% college or higher	Single item (good health/poor health)	Internet use for health information seeking (yes/no)
Nakagomi et al 2022 [66] (Japan)	To examine the association between internet use and subsequent health and well-being	Longitudinal; Japan Gerontological Evaluation Study (3 waves: 2013, 2016, 2019); self-administered questionnaires	N=3903 older adults aged ≥65; mean age 71.34 (SD 4.65) years; 62.97% women; education: 68.07% had ≥10 years, 31.92% had ≤9 years	Single item (excellent/good vs others)	Frequency of internet use (4-point scale: not at all/a few times a month/a few times a week/almost every day)
Sims et al 2016 [67] (United States)	To examine the association between ICT use and well-being among the oldest-old	Cross-sectional; online/telephone survey Socioemotional selectivity theory and biological models of aging	N=445 older adults aged ≥80 years; mean age 84 (SD 3) years; 64% women; 45% had more than high school education; 26% non-White	Single item (5-point scale: 1 “excellent” to 5 “poor”)	ICT use: number of ICT devices/applications used (3-point scale: 0 “none,” 1 “one,” 2 “two or more”); motivation for ICT use: 2 items (connect with family/friends; learn new information/skills), each rated on a 5-point scale (1 “strongly disagree” to 5 “strongly agree”)
Swed et al 2020 [68] (United States)	To examine the association between internet use and SRH among older military veterans	Cross-sectional; National Survey of Veterans (2010 wave); mail questionnaire	N=8539 older adults aged ≥60 years; mean age 61.02 (SD 0.02) years; 91.5% men; education: 5.4% <high school, 26% high school, 30% some college, 27% college degree, 11.7% professional degree; 80.6% White, 10.9% Black, 4.8% Hispanic, 3.7% other	Single item (3-point scale: “fair/poor,” “good/very good,” “excellent”)	Frequency of internet use (4-point scale: daily/once a week, not daily/once a month-once per year/does not use the internet)
Tavares 2020 [69] (17 European countries and Israel)	To examine the association between internet use and SRH, and whether this association varies across countries with different levels of eHealth policy development	Cross-sectional; Survey of Health, Ageing and Retirement in Europe (SHARE), wave 6 Social ecological model (Dahlgren and Whitehead 1991 [70])	N=66,279 adults aged ≥50 years from 18 countries; mean age 67.93 years; 46.8% men; mean education 10.83 years	Single item (5-point scale: 1 “poor health” to 5 “excellent health”)	Internet use in the past 7 days (yes/no)
Wang et al 2020 [71] (China)	To examine the association between internet use and health-related outcomes and whether individual cognitive ability moderates this association	Longitudinal; Chinese General Social Survey (2012 and 2015 waves)	2012 wave: N=2821 older adults aged ≥60 years; mean age 69.10 (SD 7.20) years; 56% men; mean education 2.40 (SD 1.27); range 1 (“no education”) to 7 (“graduate and above”). 2015 wave: N=3185 older adults aged ≥60 years; mean age 69.39 (SD 7.46) years; 49% men; mean education 2.39 (SD 1.26); range 1 (“no education”) to 7 (“graduate and above”)	Single item (5-point scale: 1 “relatively poor” to 5 “very healthy”)	Frequency of internet use (5-point scale: 1 “never” to 5 “very frequently”)
Wei and Guo 2023 [72] (China)	To examine the association between smartphone use and health-related outcomes	Cross-sectional; survey; face-to-face interviews	N=1110 older adults aged ≥55 years; age category (coded): mean 2.27 (SD 0.73); range 1 (“55-64”), 2 (“65-74”), 3 (“>75”); 47.02% men; mean education 1.71 (SD 0.91); range 1 (“primary school or below”) to 4 (“junior high or above”); 44% lived in urban areas	Single item (3-point scale: 1 “poor,” 2 “average,” 3 “good”)	Smartphone use (yes/no)
Wei et al 2022 [73] (China)	To examine the association between WeChat use and SRH	Cross-sectional; China Health and Retirement Longitudinal Study (2018 wave) Social participation theory (Lian et al 1999 [74])	N=5442 older adults aged ≥60 years; mean age 69.34 (SD 7.23) years; 51% women; mean education 3.00 (SD 1.91); range 1 (“illiterate”) to 11 (“PhD”)	Single item (5-point scale: 1 “very good” to 5 “very bad”)	WeChat use (use/do not use)
Wen et al 2023 [75] (China)	To examine the association between internet use and health-related outcomes	Cross-sectional; China Health and Retirement Longitudinal Study (2018 wave)	N=13,474 middle-aged and older adults; mean age 61.50 (SD 9.30) years; 51.57% women; education: 87.45% below middle school, 10.47% high school/vocational training, 1.81% above high school; 37.39% lived in urban areas	Single item (3-point scale: 1 “positive,” 2 “general,” 3 “negative”)	Internet use (yes/no)
Xiaobing and Meng 2022 [76] (China)	To examine the association between internet use and community participation, including the mediating role of SRH	Cross-sectional; China Health and Retirement Longitudinal Study (2016 wave) Displacement theory	N=8856 older adults aged ≥60 years; mean age 70.20 (SD 7.55) years; 51.63% women; mean education 3.29 (SD 1.31); range 1 (“illiterate”) to 6 (“above college”)	Single item (5-point scale: “very unhealthy” to “healthy”)	Frequent internet access (yes/no) Frequency of internet use (5-point scale: 0 “rarely” to 4 “always”) Internet as a primary source of information (yes/no)

^a^SRH: self-rated health.

^b^ICT: information and communication technology.

**Table 2:** Main results of included studies on internet use and self-rated health (SRH).

Authors (country)	Level of SRH	Level of internet usage	The impact of internet use on SRH	
			Positive/negative/NS^a^	Mediators, moderators, and heterogeneity across groups
Chen et al 2022 [46] (China)	39% reported being healthy	22% used the internet	Positive	Mediated by cultural engagement
Chopik 2016 [48] (USA)	Moderate-high level; mean 3.26, SD 1.05	Low level (social technology use for social connection); mean 1.37, SD 1.42	Positive	Mediated by reduced loneliness
Ding et al 2023 [49] (China)	74% reported good health	24% had internet access	Positive	Stronger among vulnerable groups (eg, difficulties in ADL^b^, no social participation, or no spouse)
Duplaga 2021 [50] (Poland)	67.9% reported at least satisfactory health	51.1% did not use the internet, 7.9% used the internet a few times a month or less, 14.8% used the internet a few times a week, and 26.2% used the internet every day	Negative	N/A^c^
Falk Erhag et al 2019 [51] (Sweden)	11.97% excellent; 36.18% very good; 35.56% good; 14.35% moderate; 1.94% poor	66.10% reported using the internet daily	Positive (minor effect compared to health-related variables)	N/A
Fjell et al 2020 [52] (Norway)	Moderate level; mean 2.84, SD 0.88	44% used the internet	Positive	N/A
Gracia and Herrero 2009 [53] (Spain)	83.2% reported good health	17.3% used the internet	Positive (the relationship between internet use and SRH disappeared once social class was considered)	N/A
Jeon and Choi 2024 [54] (Korea)	Moderate level; mean 2.44, SD 0.81	Internet use for interpersonal communication: high level; mean 2.24, SD 0.74 Internet use for instrumental purposes: moderate level; mean 3.22, SD 2.22	Positive (the impact of internet use for instrumental purposes was stronger than the impact of internet use for communication purposes on SRH)	N/A
Kim et al 2020 [55] (USA)	Moderate-high; mean 3.4, SD 0.02	Information technology—personal tasks: 32% used the internet for personal tasks; 44% shopped groceries/personal items; 44.2% paid bills/banking; 17.6% ordered/refilled prescriptions Information technology—health-related information: 15.9% contacted medical providers; 11.8% handled medical/health insurance matters; 36.2% gathered information about health conditions Communication technology: 65.5% emailed/texted most days; 28.4% some days; 15.1% rarely	Positive for overall ICT^d^; NS for information technology only; NS for communication technology only.	N/A
Koopman-Boyden and Reid 2009 [56] (New Zealand)	N/A	51% used the internet	Positive	N/A
Lee and Jang 2022 [57] (Korea)	Young-old: moderate-high level; mean 2.61, SD 0.80 Old-old: moderate-high level; mean 2.44, SD 0.70	Young-old: moderate to moderate-high level of increase in internet use: social networking and information-sharing services (mean 3.24, SD 0.58), social participation (mean 2.89, SD 0.59), daily services (mean 3.20, SD 0.59), search/email/content services (mean 3.26, SD 0.57) Old-old: moderate level of increase in internet use: social networking and information-sharing services (mean 2.98, SD 0.44), social participation (mean 2.87, SD 0.51), daily services (mean 2.98, SD 0.42), search/email/content services (mean 3.01, SD 0.42)	Positive	Age differences: positive association between SRH and social networking/information-sharing services and online daily services in both groups; social participation services and search/email/content services were positively associated with SRH only among the old-old group
Lee et al 2018 [58] (United States)	Moderate-high level; mean 3.2, SD 0.03	Communication technology: 15.7% emailed/texted some days; 26.8% most days Information technology (personal tasks): 31.7% used the internet for personal tasks Health matters: 26.5% used the internet for health matters	Positive for communication technology (significant only when used most days); NS for information technology and health matters	N/A
Li et al 2023 [59] (China)	N/A	Sample 1: moderate level of frequency of internet use; mean 3.03, SD 1.87 Sample 2: mean 0.19, SD 0.71; 6.7% used the internet in the last month	Positive among both samples	N/A
Liu et al 2023 [60] (China)	Moderate-high level; mean 3.079, SD 1.021	14% used the internet Low frequency of internet usage; mean 0.387, SD 0.982	Positive	Mediated by social engagement; stronger contribution among those with children (vs without children)
Liu et al 2022 [61] (China)	Moderate level; mean 2.97, SD 1.07	23.1% had access to the internet	Positive	Mediated by social support (relatives and friends); stronger among male older adults, younger older adults, and rural older adults
Lyu and Sun 2021 [62] (China)	53.47% reported that they were healthy	12.87% used the internet	Positive	Mediated by social capital
Millar et al 2020 [63] (United States)	85.2% reported good health	69.96% used the internet to seek health information	NS	N/A
Nakagomi et al 2022 [66] (Japan)	87.8% reported excellent/good health	47.7% not at all; 14.7% a few times a month; 13.5% a few times a week; 24.2% almost every day	Positive (modest link; significant only for almost every day internet use); NS for a few times a month/a few times a week	N/A
Sims et al 2016 [67] (United States)	Moderate level; mean 2.87, SD 0.87	Mean number of devices/applications used 1.23, SD 0.83 Moderate-high level of using technology to connect with family and friends; mean 3.66, SD 1.28 Moderate level of using technology to learn new information and skills; mean 2.61, SD 1.44	Positive	Mediated by using technology to learn new information; not mediated by using technology to connect with family and friends
Swed et al 2020 [68] (United States)	72.4% had good/very good/excellent health	48.7% used the internet daily; 16.6% used it once a week (not daily); 5.6% used it once a month-once per year; 29.1% did not use the internet	Positive	N/A
Tavares 2020 [69] (17 European countries and Israel)	11.42% poor; 28.56% fair; 35.61% good; 17.62% very good; 6.78% excellent	48.4% used the internet in the previous 7 days	Positive	Stronger in less eHealth-developed countries; not beneficial for older adults with low levels of health
Wang et al 2020 [71] (China)	Moderate-high level in both waves; mean 3.01, 3.18, SD 1.05, 1.06 (2012 and 2015, respectively)	Low level in both waves; mean 1.20, SD 0.78 and mean 1.35, SD 0.93 (2012 and 2015, respectively)	NS	Not moderated by individual cognitive ability
Wei and Guo 2023 [72] (China)	Moderate-high level; mean 2.377, SD 0.760	57% used a smartphone	Positive	Stronger among urban residents and among adults aged >75 years; mediated by performance expectations and individualized needs
Wei et al 2022 [73] (China)	Moderate-high level; mean 3.12, SD 1.04	5% used WeChat	Positive	Stronger among younger-older adults (<70 years); positive effects among both older men and women
Wen et al 2023 [75] (China)	23.85% positive health; 49.49% general health; 26.65% negative health	24.32% used the internet	Positive	Significant among older adults living in both rural and urban settings
Xiaobing and Meng 2022 [76] (China)	8.34% very unhealthy; 39.08% relatively unhealthy; 34.16% average; 14.72% relatively healthy; 3.69% very healthy	Internet use: 88.91% did not use the internet; internet use frequency: 83.68% never; 4.98% rarely; 4.93% sometimes; 4.43% often; 1.98% always**;** internet as an information source: 4.53% used the internet as a source of information	Positive for frequent internet use, and for using the internet as a source of information	SRH mediated the relationship between internet use and community engagement
Yang et al 2020 [77] (USA)	Young and middle-aged: 26.8% excellent; 37.9% very good; 25.0% good; 8.2% fair; 2.1% poor Older adults: 16.1% excellent; 33.3% very good; 33.4% good; 13.6% fair; 3.8% poor	Young and middle-aged: 85.6% daily; 6.1% use but not daily; 8.3% never Older adults: 43.5% daily; 12.4% use but not daily; 44.1% never	Positive	Stronger among older adults than among young and middle-aged adults

^a^NS: not significant.

^b^ADL: activities of daily living.

^c^N/A: not applicable.

^d^ICT: information and communication technology.

## Results

### Overview

**Figure 1:**
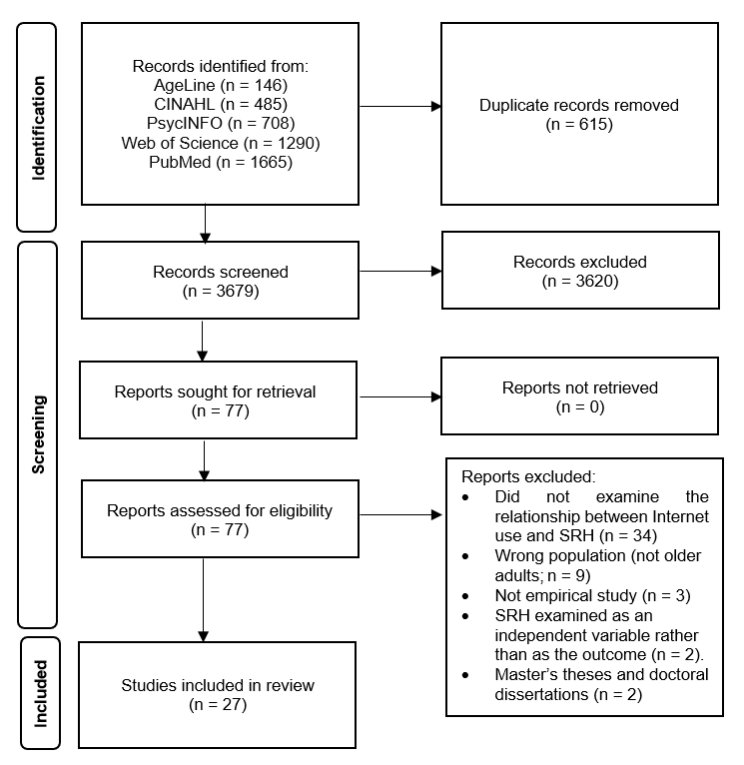
PRISMA (Preferred Reporting Items for Systematic Reviews and Meta-Analyses) 2020 flow diagram of study selection. SRH: self-rated health.

The study selection process is shown in Figure 1. Searches of 5 databases identified 4294 records (PubMed, n=1665; Web of Science, n=1290; PsycINFO, n=708; CINAHL, n=485; AgeLine, n=146). After removing 615 duplicates, 3679 records were screened by title and abstract, and 77 full-text reports were assessed for eligibility. A total of 27 studies were included. The 50 excluded full-text reports were primarily excluded because they did not examine the internet use-SRH relationship (n=34), involved an ineligible population (n=9), were not empirical studies (n=3), treated SRH as an independent variable (n=2), or were theses/dissertations (n=2).

### Characteristics of Included Studies

#### Overview

Table 1 provides an overview of the included studies and summarizes the following elements: study aims; publication year and country; study design and theoretical framework (where applicable); sample characteristics; and measures of SRH and internet use. Overall, the evidence base comprised predominantly cross-sectional, quantitative survey studies and included relatively limited use of explicit theoretical frameworks. Across studies, SRH was consistently assessed using a single-item measure (with varying response scales), whereas internet use was operationalized across multiple dimensions, including access/use (yes/no), frequency of use, and purpose-specific or domain-based measures.

#### Aims of the Studies

Across nearly all included studies (26/27), internet use was examined as the independent variable and SRH as the outcome. In 1 study, SRH was examined as a mediator in the association between internet use and community participation [76].

#### Publication Years and Geographical Distribution

Most included studies (n=22) were published between 2019 and 2024. Three studies were published between 2016 and 2018 (2016: n=2; 2018: n=1), and 2 studies were published in 2009. Studies were most frequently conducted in China (n=11) and the United States (n=7). Two studies were conducted in Korea, and 1 study each was conducted in Poland, Sweden, Norway, Spain, New Zealand, and Japan. One cross-national study analyzed data from 17 European countries and Israel [69].

#### Study Designs and Data Collation

All included studies were quantitative and relied on survey or structured questionnaire data. **Notably,** no qualitative or mixed methods studies met the inclusion criteria. Several studies drew on the same underlying survey datasets: 3 studies used the Chinese General Social Survey (CGSS) [46,61,71], 5 studies used the China Health and Retirement Longitudinal Study (CHARLS) [49,59,60,75,76] (including 1 study that combined CHARLS with an additional dataset [59]), and 2 studies used the National Health and Aging Trends Study [55,58]. Two studies used longitudinal designs [66,71], while the remainder were cross-sectional. Specifically, Nakagomi et al [66] (2022) analyzed 3 waves of the Japan Gerontological Evaluation Study (2013, 2016, and 2019), and Wang et al [71] (2020) analyzed 2 waves of the CGSS (2012 and 2015).

#### Sample Characteristics

Most included studies focused on community-dwelling populations. Although the review targeted older adults, 4 studies drew on datasets that included younger age groups in addition to older adults (eg, samples defined as ≥45, ≥50, or ≥35 years), while still reporting results specifically for older adults as a separate group [50,60,63,69]. In 1 study, older adults were explicitly compared with a younger group (18-64 years vs ≥65 years) [77]. One study focused on older cancer survivors [58], and 1 examined older military veterans [68].

Sample sizes varied widely. Four studies included fewer than 1000 participants (range 233-709) [48,52,53,67]. Most studies (n=18) used large samples of 1000-10,000 participants (range 1000-9434), including 1 study that analyzed 2 samples (n=598 and n=9434) [59]. Five studies used very large samples of >10,000 participants (range 13,474-82,014) [49,60,69,75,77].

#### Theoretical Framework

Most included studies (21/27) did not report an explicit theoretical framework. Six studies referenced a theory or conceptual model: Chen et al [46] drew on activity theory; Wei et al [73] drew on social participation theory; Sims et al [67] referenced socioemotional selectivity theory and biological models of aging; Tavares [69] used a social ecological model; Millar et al [63] drew on Paasche-Orlow and Wolf’s model and Gewald and Rockmann’s model; and Xiaobing and Meng [76] drew on displacement theory. Overall, these findings indicate that the association between internet use and SRH has largely been examined in an atheoretical manner.

#### Measurements

Overall, SRH was measured consistently across studies using a single-item indicator, although response scales varied. In contrast, internet use was assessed across 3 main dimensions: access/use (yes/no), frequency (ordinal scales), and purpose or domain of use (eg, social, instrumental, or health-related).

#### SRH Measurements

All included studies assessed SRH using a single item, typically phrased as a global self-assessment of health. Response scales varied: 8 studies used dichotomous response options (eg, unhealthy/healthy; poor/good health; or satisfied/dissatisfied with health) [46,49,50,53,56-63,66]. Three studies used 3-point scales [68,72,75], 1 study used a 4-point scale [57], and 15 studies used 5-point scales [48,51,52,54,55,58-61,67,69,71, 73,76,77].

#### Internet Use Measurements

Measures of internet use varied substantially across studies. Nine studies measured internet use/access using a single binary indicator (eg, yes/no; user/nonuser; or use in the past 7 days) [46,49,52,53,56,61,62,69,75]. Nine studies assessed frequency of internet use using ordinal response scales ranging from 3-point to 7-point measures (including 3-point [77], 4-point [50,66,68], 5-point [71], and 7-point [51]). In addition, 3 studies used more than 1 internet-use measure [59,60,76]: Liu et al [60] included both a binary internet use indicator (yes/no) and a frequency measure (4-point scale); Xiaobing and Meng [76] included multiple internet-use indicators, including indicators of access/use, a measure of use frequency, and the internet as a primary source of information, and Li et al [59] used different frequency measures across samples (a 5-point scale in sample 1 and a 4-point scale in sample 2).

Several studies operationalized internet use in more specific ways, including socially oriented technology use [48], interpersonal communication and instrumental internet use [54], changes in internet use across multiple online domains [57], and internet use for health information seeking [63]. Finally, some studies assessed broader information and communication technology (ICT) use rather than internet use alone, including ICT indicators or indices and counts of devices or applications and related motivations [55,58,67]. Some studies also used platform- or device-specific measures, such as smartphone use or WeChat use [72,73].

### Main Results of Included Studies on Internet Use and SRH

#### Overview

Table 2 summarizes the main findings of the included studies. It reports levels of SRH and internet use, the direction and significance of the association between internet use and SRH, and, where available, mediators and heterogeneity across subgroups. Overall, Table 2 indicates that most studies report a positive association between internet use and SRH. However, the magnitude and robustness of this association vary depending on the type of internet use examined and the characteristics of the study population.

#### Levels of SRH

Across studies, SRH levels were reported either as mean scale scores or as proportions. In 12 studies reporting mean SRH scores, the average level of SRH was generally moderate to moderately high [48,52,54,55,57,58,60,61,67,71-73]. In studies reporting proportions, the share of participants reporting good to excellent health was often high (approximately 75% to the mid-80% range) [49,51,53,63,68], with an even higher proportion reported by Nakagomi et al [66] (87.8%). Lower proportions were also reported in some studies, including 53.47% reporting healthy status in the study by Lyu and Sun [62] and 39% reporting healthy status in the study by Chen et al [46].

#### Levels of Internet Use

Overall, reported levels of internet use varied widely across studies, partly because internet use was operationalized in different ways (eg, binary use, frequency categories, or mean/index scores). Seven studies reported a relatively low prevalence of internet use, generally around 11%-24% [46,49,60-62,75,76]. In contrast, 4 studies reported substantially higher prevalence, with around 44%-57% of participants using the internet (or using a smart device) [52,56,69,72]. In studies reporting frequency-based measures, daily or near-daily use also varied considerably: 2 studies reported that roughly 25% of participants used the internet daily or almost every day [50,66], whereas 2 other studies reported higher daily-use rates including 48.7% [68] and 66.1% [51].

Several studies also reported purpose- or domain-specific levels of internet use. Social or communication-oriented use was reported as a low mean level of socially oriented technology use (mean 1.37, SD 1.42) [48], moderate to high engagement in communication technology in some samples (eg, emailing/texting most days, 65.5%) [55], and lower engagement in others (eg, emailing/texting some days, 15.7%, and most days, 26.8%) [58]. In 1 study assessing online purposes, interpersonal communication use was reported (mean 2.24, SD 0.74), alongside instrumental use (mean 3.22, SD 2.22) [54], and another study reported moderate to moderately high levels of change across multiple online domains (approximate means 2.87-3.26 across domains) [57]. Health-related internet use also varied: in 1 study, specific health-related online activities ranged from 11.8% to 36.2% [55], whereas another study reported 69.96% using the internet for health information seeking [63]. Finally, platform- or device-specific use was generally low for WeChat (5%) [73] and higher for smartphone use (57%) [72].

#### The Association Between Internet Use and SRH

The majority of included studies (24/27) reported a positive association between internet use and SRH, indicating that older adults who used the internet tended to report better SRH than nonusers [46,48,49,51-62,66-69,72,73,75-77]. Consistent with this pattern, studies assessing frequency-based measures generally suggested that more frequent internet use was associated with better SRH [54,57,59,66-68,76,77]. Purpose-specific measures showed positive associations in several contexts, including socially oriented technology use and communication-related use [48,54,57,58], and instrumental use, such as accessing information and services, in some studies [54,57]. One study also reported that combined ICT use was associated with better SRH [55].

At the same time, findings were not uniform. One study reported a modest association that was significant primarily for near-daily internet use [66], and another found that communication technology was positively associated with SRH only when used most days [58]. One study reported that the contribution of internet use to SRH was minor compared with health-related factors, such as chronic or psychiatric conditions [51], and another found that the association was attenuated after accounting for social class [53]. Two studies reported no significant association between internet use and SRH [63,71], and 1 study reported a negative association [50]. In addition, nonsignificant results were reported for specific types of health-related internet use in some studies [58,63].

#### Mediating Factors in the Relationship Between Internet Use and SRH

Six of the included studies (6/27) examined mediating pathways linking internet use with SRH. Several of these studies highlighted social pathways: cultural engagement [46], social support from relatives and friends [61], and social engagement [60] were each reported as mediators of the association between internet use and SRH. Loneliness was also identified as a mediator in 1 study, with socially oriented technology use associated with lower loneliness, which in turn was linked to better SRH [48]. In addition, 1 study reported that using technology to learn new information and skills mediated the association between ICT use and SRH, whereas using technology to connect with family and friends did not show a mediating effect [67]. Finally, 1 study identified performance expectations and individualized needs as mediators of the association between smart device use and SRH [72].

#### Heterogeneity Across Subgroups in the Association Between Internet Use and SRH

Studies examining subgroup differences in the association between internet use and SRH reported heterogeneous findings. With respect to age, 1 study comparing younger and older adults reported a stronger association between smartphone use and SRH among older adults than among younger and middle-aged adults [77]. In addition, age-stratified analyses in 1 study of internet use domains showed that social networking and information sharing and daily services were positively associated with SRH in both the young-old (aged 65-74 years) and old-old (aged ≥75 years) groups, whereas social participation services and search, email, and content services were positively associated with SRH only in the old-old group [57]. Other studies also suggested that the benefits of internet and social media use may be more pronounced among younger-old adults (eg, aged <70 years) [61,73].

Regarding place of residence, findings were mixed: 1 study reported a stronger benefit of internet use among rural older adults [61], whereas another reported a stronger association between smartphone use and SRH among urban older adults [72]. A third study found no differences between urban and rural settings [75]. For gender, 1 study reported a stronger benefit of internet use among older men than older women [61], whereas another study found no gender differences in the association [73]. Evidence of heterogeneity was also reported for other population characteristics. Internet access appeared more beneficial for vulnerable groups (eg, those with difficulties in activities of daily living) and for individuals with lower social participation or without a spouse [49]. In contrast, 1 study reported that internet use was not beneficial among older adults with low levels of health [69], while also indicating a stronger association between internet use and SRH in countries with less developed eHealth contexts [69]. In 1 study, cognitive ability was examined as a moderator, but no significant moderating effect was found [71]. Finally, 1 study reported that the contribution of internet use to SRH was greater among older adults with children than among those without children [60].

## Discussion

### Principal Findings

The purpose of this scoping review was to map and characterize the existing literature on internet use and SRH among older adults, including how this relationship has been studied, what patterns of association have been reported, and which gaps remain in the evidence. Overall, the review included 27 studies, most of which (24/27) reported a statistically significant positive association between internet use and SRH among older adults. Several studies further suggested that socially oriented uses of the internet, including communication and social participation, were positively associated with SRH [48,57,58]. Evidence on potential mechanisms was more limited; however, the available findings indicate that social factors, including social support and social participation, may help explain the observed association between internet use and SRH [46,60-62]. In addition, some studies suggested that certain vulnerable groups, such as individuals with difficulties in activities of daily living, lower social participation, or without a partner, may derive greater benefit from internet use, although subgroup findings were mixed overall [49]. These findings can be interpreted in light of social participation theory, which posits that active engagement in social activities, whether in-person or virtual, can enhance well-being and contribute to better perceived health in later life. Similarly, social capital theory highlights the importance of social networks and the resources they provide, including emotional and instrumental support [78]; these resources may be expanded or sustained through internet use and, in turn, relate to SRH. Beyond these perspectives, as a possible theoretical interpretation, the findings may also be viewed through the lens of socioemotional selectivity theory [79], which suggests that as individuals age, they increasingly prioritize emotionally meaningful goals and relationships. From this perspective, socially oriented internet use, including communication with family and friends or participation in online social activities, may be particularly relevant to older adults’ SRH because it supports emotionally salient connections that are central to well-being in later life.

At first glance, these findings suggest that interventions supporting older adults in acquiring internet skills may be relevant for supporting SRH. However, a closer examination highlights persistent digital gaps. Several studies in this review reported relatively low levels of internet use, particularly in studies that included a binary indicator of internet use (eg, Chen et al [46], Liu et al [60], Lyu and Sun [62], and Xiaobing and Meng [76]). Although internet adoption among older adults has increased over the past decade [33-35], evidence continues to point to a digital divide [80,81]. Digital divide frameworks conceptualize digital inclusion as a cumulative process that involves motivation to use digital technologies, physical access, and digital skills [82]. Consistent with this perspective, the divide reflects not only age-related barriers (ie, “gray” gaps), including negative attitudes toward technology, limited digital skills, lack of interest, and experiences of ageism [33,83,84], but also broader sociodemographic and structural disparities, including lower socioeconomic resources, lower education, poorer health, and disability [34-46,48-63,66-69,71-73,75-89]. As a result, it is important that interventions address access conditions, including infrastructure, devices, and affordability, as well as guidance and support that strengthen digital skills and enable safe and meaningful use. Such a combined approach may help reduce inequalities in opportunities for social connection, access to information and services, and potential health-related benefits.

Indeed, most studies in the review reported a positive association between internet use and SRH; however, 1 study reported a negative association [50], and 2 studies reported no significant relationship [63,71]. Duplaga [50] found that more frequent internet users were more likely to rate their health as poor, possibly reflecting a more critical self-appraisal associated with exposure to health-related content. It is therefore important to acknowledge that internet use may also entail potential risks for older adults. One concern is exposure to online health misinformation, which can lead to confusion, mistrust in health professionals, and potentially harmful health behaviors [90,91]. Additional risks include digital fatigue and information overload, which may contribute to cognitive strain and mental exhaustion [91-93], as well as cybersecurity threats, such as scams, fraud, and privacy violations, which can increase stress and reduce trust in digital technologies [94,95]. Together, these considerations highlight the need for digital literacy programs and supportive interventions to help ensure that internet use promotes, rather than undermines, older adults’ health and well-being. Finally, the absence of a positive association between internet use and SRH in some studies may reflect background and contextual factors, such as socioeconomic resources (eg, income and education), cognitive ability, and the inherently subjective and multidimensional nature of SRH, which may be less responsive to behavioral influences such as internet use [63,71].

Despite the importance of the results of the included studies, we identified several research gaps that limit our understanding of the relationship between internet use and SRH. First, it is important to note that a significant portion of the studies included in this review (25/27) were based on a cross-sectional design, a common methodological approach that, by its nature, limits the ability to infer causal relationships between variables because it captures phenomena at a single point in time [96]. This methodological limitation requires careful consideration when interpreting the findings and generalizing them to dynamic or process-based relationships. Nevertheless, the included studies met most of the key criteria for assessing risk of bias in cross-sectional research, as outlined in the methodological review by Kelly et al [97], including selection (sample representativeness), exposure (measurement validity), outcome (measurement reliability), confounding (control of variables), missingness (handling missing data), selective reporting (complete results disclosure), and conflict of interest (financial or professional influence). The overall methodological quality strengthens the degree of confidence that can be placed in the review’s findings, even if conclusions must be drawn with caution. In light of the methodological challenges inherent to this design, there is a clear need to promote longitudinal and interventional studies that allow for a deeper understanding of dynamic relationships and causal pathways over time. Second, most of the studies did not apply a theoretical framework or model. Only 6 of the 27 studies referred to a theory, such as activity theory, social participation theory, or the social ecological model. The limited use of theoretical frameworks may reflect the lack of models that are specifically tailored to understanding the relationship between internet use and SRH in older adults. Without a guiding framework, it becomes more difficult to interpret the processes underlying this relationship, including possible mediators and moderators. Therefore, future research should aim to develop or adopt theoretical models that can offer clearer conceptual guidance for investigating this association. Third, regarding internet use, the majority of the studies focused primarily on examining use or frequency of use without reference to the purpose of internet use. Therefore, further studies should focus on understanding how the different purposes for which the internet is used may affect older adults’ SRH. Finally, this review included only studies from industrialized countries, highlighting the need for future research in developing countries and low- and middle-income countries. In these contexts, internet access is often more limited, and older adults may encounter unique barriers such as lower levels of digital literacy, limited technological infrastructure, and different social and health care systems [98]. In addition, sociocultural factors such as norms surrounding aging, family roles, and attitudes toward technology may also influence patterns of internet use and its impact on SRH [99,100]. Therefore, findings from low- and middle-income countries may differ substantially from those in high-income countries and warrant focused, context-sensitive investigation.

### Limitations

This scoping review has several limitations. Although the search strategy followed established guidelines and covered 5 major electronic databases, supplemented by Google Scholar searches and reference list screening, relevant studies published in sources not indexed in these databases may have been missed. In addition, the review was restricted to English-language publications; therefore, relevant studies published in other languages may have been excluded. Finally, the review included only peer-reviewed journal articles and did not incorporate gray literature, despite evidence that gray literature can provide valuable insights in systematic and scoping reviews [101].

### Conclusions and Implications for Practice and Policy

This scoping review mapped 27 studies examining the association between internet use and SRH among older adults. Most studies reported a positive association between internet use and better SRH, although the magnitude and consistency of this relationship varied depending on how internet use was measured and on the characteristics of specific subgroups. Overall, the findings suggest that the relationship between internet use and SRH depends substantially on the purpose of use, with socially oriented uses such as communication and social participation appearing particularly relevant and potentially operating through mediating social pathways, including greater perceived support and reduced loneliness. However, because the evidence base is dominated by cross-sectional studies, longitudinal and intervention research is needed to strengthen causal inference, strengthen and extend the evidence on mediating mechanisms, which have been examined in a relatively small number of studies and mainly through social factors, and on moderating mechanisms, and to clarify for whom and under what conditions internet use contributes to SRH.

From a practical standpoint, the findings support advancing digital inclusion as part of healthy aging policy by providing age-tailored training that promotes safe and meaningful use, especially uses that facilitate social connection, access to information and services, and community participation. Digital support should be integrated into health and social care through community-based initiatives and digital navigators who can provide hands-on guidance and ongoing assistance. In addition, improving digital inclusion may also benefit health and social care systems by facilitating timely access to services and support and potentially contributing to greater system efficiency [102]. This may also help reduce health disparities among older adults [102]. At the same time, maintaining nondigital options across service points and information channels is important to prevent exclusion of those who do not use the internet or struggle to do so. These recommendations align with framing digital inclusion as a social determinant of health rather than merely a technological issue [102]. Finally, there remains a notable gap in evidence from low- and middle-income countries, where sociocultural contexts and system-level conditions may shape patterns of internet use and its health implications [99,100]. In these settings, and among socioeconomically disadvantaged groups within high-income countries, digital inclusion efforts may also need to address structural barriers such as affordability, access to connectivity, and the availability of accessible online services tailored to older adults.

## Data Availability

The datasets used during this study are available from the corresponding author on reasonable request.

## Appendix

Multimedia Appendix 1 PRISMA-ScR checklist.

Multimedia Appendix 2 PRISMA-S checklist.

Multimedia Appendix 3 Search strategies.

## Abbreviations

CGSS: Chinese General Social Survey
CHARLS: China Health and Retirement Longitudinal Study
ICT: information and communication technology
JBI: Joanna Briggs Institute
PRISMA-S: Preferred Reporting Items for Systematic Reviews and Meta-Analyses extension for Searching
PRISMA-ScR: Preferred Reporting Items for Systematic Reviews and Meta-Analyses extension for Scoping Reviews
SRH: self-rated health
