# Supplementary material for: Internet Use and Self-Rated Health Among Older Adults: Scoping Review
Source: Interact J Med Res. 2026 Feb 19;15:e76930. doi: 10.2196/76930 (PMC12963975; doi:10.2196/76930)
Supplement: Multimedia Appendix 3 [file ijmr_v15i1e76930_app3.pdf]

Multimedia Appendix 1. Search strategies for all databases

The following search strategies were used to identify relevant studies examining Internet use and self-rated health (SRH) among older adults. Searches were conducted on February 5, 2024, and included all publications available up to that date. Searches were not restricted to the title or abstract fields and were conducted across all available fields, where applicable. Searches were limited to English-language publications. Where applicable, results were further limited to peer-reviewed journal articles.

PubMed

("Internet" OR "web" OR "online" OR "digital" OR "technology" OR "information and communication technology" OR "social media" OR "computer" OR "smartphone" OR "tablet")
AND
("self-rated health" OR "self-reported health" OR "self-assessed health" OR "perceived health" OR "subjective health")
AND
("older adults" OR "aging" OR "aged" OR "older people" OR "elderly" OR "seniors")

Filters applied: English language.

CINAHL (EBSCO)

("Internet" OR "web" OR "online" OR "digital" OR "technology" OR "information and communication technology" OR "social media" OR "computer" OR "smartphone" OR "tablet")
AND
("self-rated health" OR "self-reported health" OR "self-assessed health" OR "perceived health" OR "subjective health")
AND
("older adults" OR "aging" OR "aged" OR "older people" OR "elderly" OR "seniors")

Limiters applied: English language; Peer-reviewed.

AgeLine (EBSCO)

("Internet" OR "web" OR "online" OR "digital" OR "technology" OR "information and communication technology" OR "social media" OR "computer" OR "smartphone" OR "tablet")
AND
("self-rated health" OR "self-reported health" OR "self-assessed health" OR "perceived health" OR "subjective health")
AND
("older adults" OR "aging" OR "aged" OR "older people" OR "elderly" OR "seniors")

Limiters applied: English language; Peer-reviewed.

PsycINFO (EBSCO)

("Internet" OR "web" OR "online" OR "digital" OR "technology" OR "information and communication technology" OR "social media" OR "computer" OR "smartphone" OR "tablet")
AND
("self-rated health" OR "self-reported health" OR "self-assessed health" OR "perceived health" OR "subjective health")
AND
("older adults" OR "aging" OR "aged" OR "older people" OR "elderly" OR "seniors")

Limiters applied: English language; Peer-reviewed.

Web of Science

("Internet" OR "web" OR "online" OR "digital" OR "technology" OR "information and communication technology" OR "social media" OR "computer" OR "smartphone" OR "tablet")
AND
("self-rated health" OR "self-reported health" OR "self-assessed health" OR "perceived health" OR "subjective health")
AND
("older adults" OR "aging" OR "aged" OR "older people" OR "elderly" OR "seniors")

Indexes searched: Science Citation Index Expanded (SCI-EXPANDED) and Social Sciences Citation Index (SSCI).

Filter applied: English language.
